# Quality evaluation of virtual slides using methods based on comparing common image areas

**DOI:** 10.1186/1746-1596-6-S1-S14

**Published:** 2011-03-30

**Authors:** Slawomir Walkowski, Janusz Szymas

**Affiliations:** 1 Faculty of Computing Science and Management, Poznan University of Technology, Poznan, Poland; 2 Department of Clinical Pathology, University of Medical Sciences, Poznan, Poland

## Abstract

**Background:**

There are many scanners of glass slides on the market now. Quality of digital images produced by them may be different and pathologists who examine virtual slides on a monitor may subjectively evaluate it. However, objective comparison of quality of digital slides captured by various devices requires assessment algorithms, which will be automatically executed.

**Methods:**

In this work such an algorithm is proposed and implemented. It is dedicated for comparing quality of virtual slides which show the same glass slide captured by two or more scanners. In the first step this method looks for the largest corresponding areas in the slides. This task is realized by defining boundaries of tissues and providing the relative scale factor. Then, a certain number of smaller areas, which show the same fragments of both slides, is selected. The chosen fragments are analyzed using Gray Level Co-occurrence Matrix (GLCM). For GLCM matrices some of the Haralick features are calculated, like contrast or entropy. Basing on results for some sample images, features appropriate for quality assessment are chosen. Aggregation of values from all selected fragments allows to compare the quality of images captured by tested devices.

**Results:**

Described method was tested on two sets of ten virtual slides, acquired by scanning the same set of ten glass slides by two different devices. First set was scanned and digitized using the robotic microscope Axioscope2 (Zeiss) equipped with AxioCam Hrc CCD camera. Second set was scanned by DeskScan (Zeiss) with standard equipment. Before analyzing captured virtual slides, images were stitched and converted using software which utilizes advances in aerial and satellite imaging.

The results of the experiment show that calculated quality factors are higher for virtual slides acquired using first mentioned device (Axioscope2 with AxioCam).

**Conclusions:**

Results of the tests are consistent with opinion of the pathologists who assessed quality of virtual slides captured by these devices. This shows that the method has potential in automatic evaluation of virtual slides’ quality.

## Background

Virtual slides technology enables pathologists to store slides in computer memory and watch them on a display. However, experts who use virtual slides in their work know that their quality may be different. One of the main factors affecting this quality is a type of device used to capture and digitize slides. Each pathologist may have his or her own opinion about the quality of virtual slides coming from a particular device. The problem is that such assessment is subjective and opinions given by various experts may be different.

In this approach we try to find a method of automatic evaluation of virtual slides’ quality, where human factor in the assessment is minimal. Such a method compares quality factors calculated for virtual slides captured by different devices, but showing the same real slide. Running this method for many slides and a few scanners will allow us to say which device captures virtual slides of better quality.

## Methods

The main goal of this work is designing, implementing and testing a method which compares quality factors, automatically calculated for corresponding areas of virtual slides.

When designing our method for comparing virtual slides’ quality, several criteria were followed. First of them is relatively fast execution. Size of virtual slides in computer memory is usually big, so the method does not process the whole slide, but rather some of its areas. To achieve it, the algorithm follows another criterion: ability to deal with big digital images, often stored in hundreds of single files. The method can merge them and crop according to requested areas. The third criterion is accuracy, which means producing reliable results. This is done by finding and selecting corresponding areas, which are then evaluated. This approach allows to visually examine quality of selected corresponding areas, which is helpful when assessing method’s results.

The method is divided into the following main steps:

1. Loading virtual slides.

2. Selecting corresponding areas which quality will be compared.

3. Choosing and displaying corresponding fragments of the selected areas.

4. Calculating and aggregating quality measures for the chosen fragments.

In the first step, when loading virtual slides, we assume that they are stored in sets of graphical files of a specified structure. For the test purpose two formats were supported, dedicated for virtual slides used in the experiment. There are several differences between them, like default file names or presence of metadata XML file. Moreover, one slide may be rotated in reference to the second one. All these differences are taken into account in slide loaders, so the format and orientation of all slides in the application are common. The effect of initial virtual slides loading are thumbnails, which are in fact overviews of the whole slides. They are presented to the user in the application. Once created, thumbnail is saved to the file to increase the speed of loading the same virtual slide for the next time.

**Figure 1:**
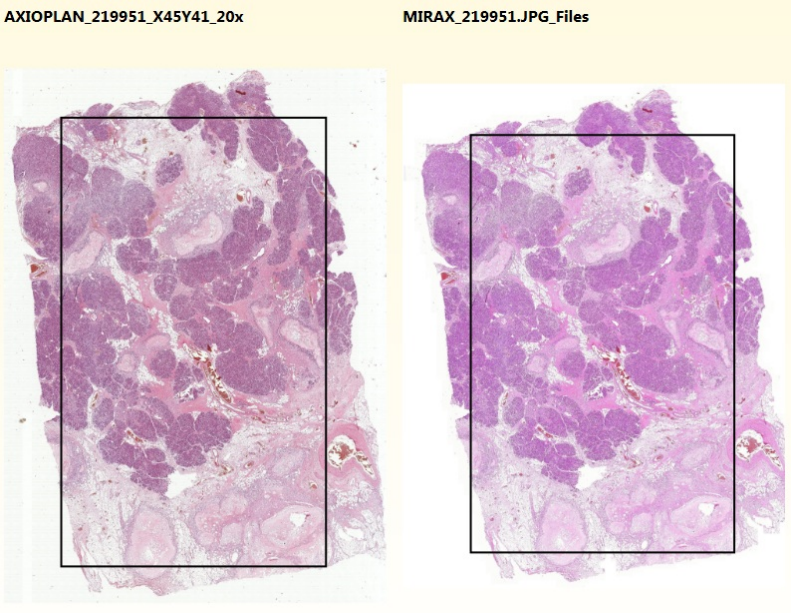
**Thumbnails of two virtual slides with selected common areas.** Thumbnails of loaded virtual slides are displayed in the application and user draws rectangles over them to mark common areas which will be taken for further analysis.

Even if two virtual slides are the effect of scanning the same glass slide using different devices, areas covered by the virtual slides may be different. Especially significant may be translations of the image. To solve this problem, in the second step of the method corresponding areas of the slides are selected by the user. Here thumbnails saved in the first step are used and user may interactively select interesting common areas by drawing rectangles which mark them out (Figure 1). Accuracy in selecting exactly corresponding areas results in more precise and reliable quality evaluation of slides’ fragments, because compared areas of virtual slides captured by different devices will be almost the same. Good way of accurate areas selection is finding two characteristic points in virtual slides and drawing rectangles between them in all loaded slides.

Area selected by the user may contain even the whole virtual slide, so processing it thoroughly could take a long time. Therefore, only some fragments of the slides are selected for the further analysis and comparison. This step can be justified by the fact that textures on a single slide are relatively monotonous and comparing quality of its fragments gives a good overview of the whole slide’s quality.

Choosing slide’s fragments is done automatically by dividing area selected by user into a grid and selecting cells with random horizontal and vertical indices. Always a certain number of cells, defined by user, is chosen (in tests 10 fragments were selected from the total number of 400 cell). Each selected cell can be described by coordinates relative to the whole selected area. These coordinates are used in each slide to cut appropriate areas from them. Resolutions of the tested virtual slides may be different, so the chosen fragments are resized to the resolution of the smallest slide. If rectangles drawn by user initially on the slides cover the same areas of the real glass slide, captured by different scanners, acquired fragments also contain the same areas. Therefore, they are taken to the further analysis.

**Figure 2:**
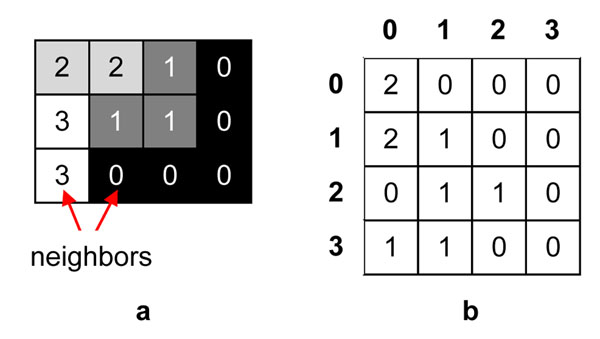
**Automatically chosen common fragments of two virtual slides**. Fragments of the selected common areas of the virtual slides are presented to the user. They can be opened in full sizes in suitable application by clicking on the links. Calculated values of contrast and entropy are displayed over the fragments.

First way of comparing the fragments is doing it manually by an expert. Software which implements this method displays thumbnails of the selected areas (Figure 2) and saves the fragments in their original resolutions to the graphical files. This allows to open them in any suitable application and visually compare some quality features, like contrast or color saturation.

**Figure 3:**
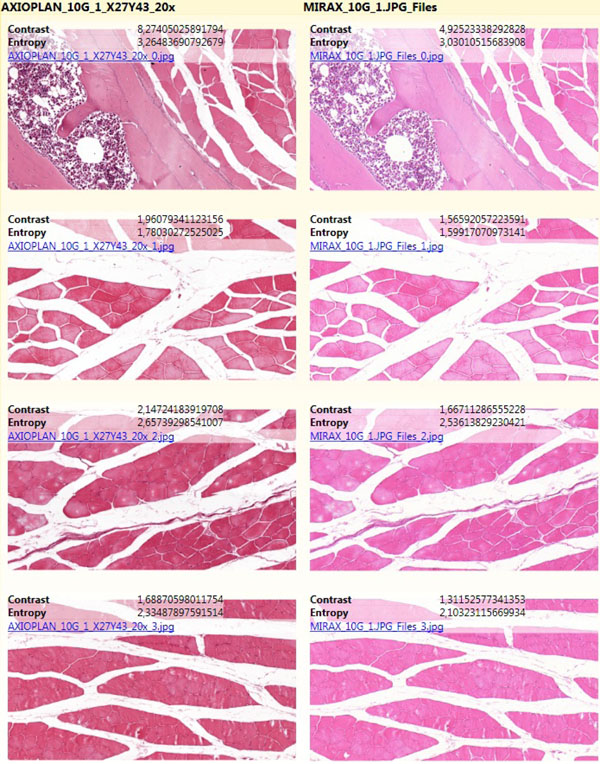
**Sample image (a) with its Gray-Level Co-occurrence Matrix for horizontal neighborhood (b)**. (a) Sample 4x3 image with pixels of 4 possible gray values (from 0 to 3); exemplary neighbors are marked; (b) Gray-Level Co-occurrence Matrix (GLCM) calculated for the sample image; numbers in the matrix denote numbers of specific pairs of neighbor pixels – row index corresponds to the value of left pixel in sample image, column index corresponds to the value of right pixel.

However, the goal of this work is to create an automatic version of virtual slides’ quality evaluation. Therefore, specific measures are calculated for the corresponding fragments selected from virtual slides. In this approach we use measures based on Gray-Level Co-occurrence Matrix (GLCM) [1][2]. The GLCM is a matrix which aggregates the distribution of co-occurring values in the image. Co-occurrence is defined using a specified offset, which determines which pixels are treated as neighbors (Figure 3a). In this work we use the smallest possible horizontal offset, which means that two consecutive pixels in a line are treated as a pair of neighbors.

GLCM aggregates the numbers of pairs of neighbor pixel values, which occur in the image. Pair of pixels (*x*, *y*) increments the cell with row index *x* and column index *y* by 1 (Figure 3b). To preserve symmetrical property of the matrix, also cell with row index *y* and column index *x* is incremented by 1. Finally, values in GLCM are divided by the sum of values in this matrix to convert the numbers to probabilities of co-occurrence of all possible pixel values.

GLCM operates on a single color channel, so each evaluated image has been converted to grayscale. This step reduces information about colors, but the main factors which decide about quality of an image, like contrast, are preserved. The number of rows and columns in the GLCM equals to the number of possible values in the grayscale. This may increase the computational complexity radically, because for 8-bit grayscale the matrix has 65536 cells. Moreover, counting every little change of pixel values in a line is probably unnecessary when assessing quality, because contrast, which is the main quality factor, depends on sharp edges which are formed by rapid pixel changes. For these reasons the grayscale depth of the chosen areas has been reduced to 5 bits.

**Figure 4:**
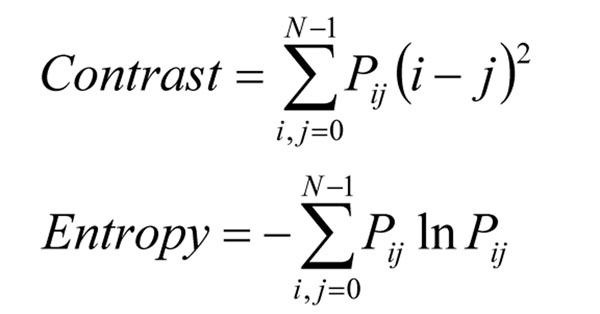
**Contrast and entropy formulas. ***N* is the size of GLCM, *P_ij_* are probabilities calculated for values in GLCM.

When GLCM is calculated for the given image, some measures can be calculated from the matrix. Haralick proposed 14 different texture measures [3], called Haralick features, which can be divided into contrast, orderliness and statistics groups. For the purpose of evaluating quality, measures from the first two groups may be useful. Because some of the measures are correlated with each other and give relatively similar results, only two measures have been selected for calculation – contrast and entropy (Figure 4).

Contrast is calculated as the sum of probabilities multiplied by the squared difference of cell indices. This formula assigns smaller weights to probabilities of co-occurrence of pixels which values do not differ significantly (especially 0 for pairs of pixels with equal values) and higher weights to probabilities of rapid changes of the pixel values in a line of the image. If we consider contrast as the sharpness of edges in an image, the contrast equation promotes images which contain many sharp edges.

The second selected measure is entropy. The formula sums up the probabilities multiplied by their natural logarithms, with reversed sign [4]. The idea of entropy comes from thermodynamics, but it is generally considered as a measure of the system’s disorganization. It is expected that entropy calculated for more complex images will be higher. When corresponding areas of virtual slides are compared, higher complexity means probably more details in an image, which results in higher quality.

**Figure 5:**
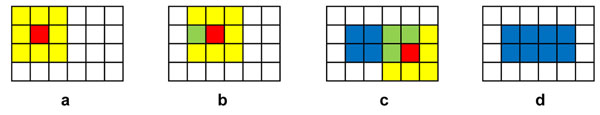
**Consecutive steps of using window when calculating measures from GLCM.** (a) 3x3 window (yellow and red pixels) in initial position; measures calculated from GLCM for this window are than saved in corresponding matrix in the position denoted by red pixel; (b) Next position of the window; green pixel is a pixel for which measures have been saved and it is again taken for the calculations in the window; (c) Last position of the window; (d) Blue pixels denote positions for which measures were calculated.

**Figure 6:**
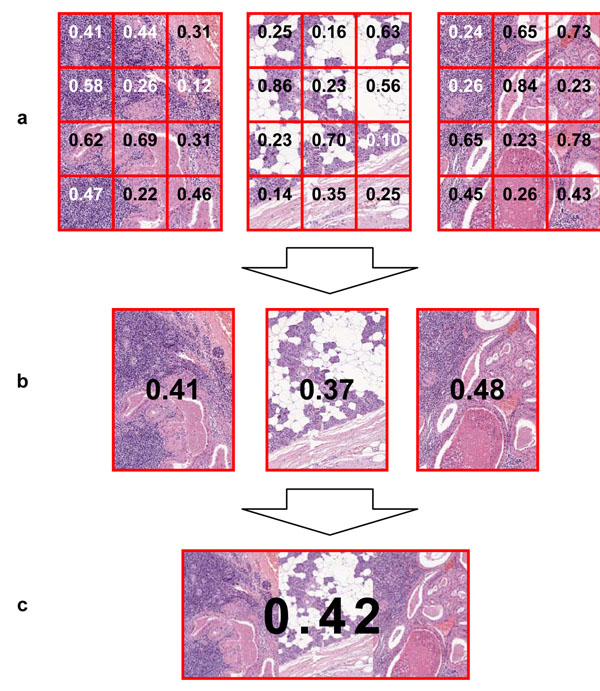
**Aggregation of measures calculated for fragments of a single virtual slide**. (a) Measures are calculated for window which moves over each fragment; window positions are in fact overlapping, which is not visible in this figure; (b) Measures are aggregated for each fragment; (c) Measures are aggregated for all selected fragments of a single virtual slide.

Single GLCM is generally calculated only for small images which horizontal and vertical sizes do not exceed a dozen of pixels. Chosen fragments of virtual slides may be much bigger, exceeding a thousand of pixels in a single dimension. In such a case, GLCM is calculated many times for a small square window of pixels which moves through an image (Figure 5). In the experiment, size of the window was set to 9. Calculated measures of the matrices are then saved in the centers of window positions (Figure 6a). Aggregation of results for a single image is done by calculating the arithmetic mean of measures from the values calculated for each window position (Figure 6b). At this stage aggregated measures can be compared with the measures calculated for corresponding fragments selected from another virtual slides. Higher value denotes higher quality factor for a given fragment.

Finally, because sizes of chosen fragments are equal, the result for the whole slide is calculated as the arithmetic mean of aggregated measures for single fragments (Figure 6c). The final value can be compared with analogous values calculated for other virtual slides, which were used in the test for a single glass slide.

## Results

The described method of quality evaluation was tested on two sets of 10 slides scanned using two different devices. One set of slides was scanned and the images were captured and digitized by using a robotic microscope Axioscope2 (Zeiss) equipped with AxioCam Hrc CCD camera (called Axioplan slides in this paper). Second set of the same glass slides was made with the use of commercially available DeskScan (Zeiss) with standard equipment (called Mirax slides). For stitching and conversion we used software based on and utilizing advances in aerial and satellite imaging.

In each test two virtual slides, containing the image of the same glass slide, were loaded. Then, we selected corresponding areas, which quality was examined in further steps. After the quality evaluation process was finished, the results were presented on the display (Figure 2). Firstly, we ensured that corresponding fragments contain the same areas of the whole slides. Then, the results were analyzed and summarized.

Final results are presented in Tables 1 and 2. They contain comparisons for 8 glass slides, because in two cases virtual slides appeared to be of totally different resolutions and contain different areas of the glass slide. Table 1 shows the aggregated contrast calculated for the corresponding fragments of the slides. Analogously, Table 2 presents the values of aggregated entropy.

**Table 1:** Contrast values for tested virtual slides

Test Number	Axioplan	Mirax
1	3.640	2.534
2	5.198	3.588
3	3.774	3.304
4	7.400	5.814
5	5.013	4.687
6	4.097	2.531
7	3.397	3.163
8	4.735	3.513

Values of contrast, returned by the tested method, calculated in each test for both Axioplan and Mirax virtual slides, which contain common area of the same glass slide.

**Table 2:** Entropy values for tested virtual slides

Test Number	Axioplan	Mirax
1	2.866	2.605
2	3.574	3.195
3	4.245	3.923
4	4.180	3.866
5	4.037	3.731
6	4.044	3.461
7	3.834	3.765
8	4.714	4.198

Values of entropy, returned by the tested method, calculated in each test for both Axioplan and Mirax virtual slides, which contain common area of the same glass slide.

## Discussion

We can see that for all slides values of contrast for Axioplan slides are higher than corresponding values for Mirax slides. In one or two cases the difference is relatively low, but in most cases it is significant. Comparison of entropy for the same set of slides is similar – in all 8 tests entropy of Axioplan slides is higher than entropy of Mirax slides. This means that the quality factors returned by the method described in this paper are higher for Axioplan slides than Mirax slides. Therefore, in this test the set of Axioplan slides is selected by this method as the set of virtual slides of better quality.

These results are consistent with opinions of the pathologists, who claim that quality of virtual slides captured by robotic microscope Axioscope2 equipped with AxioCam camera is better than quality of corresponding virtual slides scanned by DeskScan with standard equipment. The difference is said to be bigger on higher magnification levels, which were examined in these tests. Common fragments, selected for analysis by this method, can be also compared visually, even by non-specialists. Conclusions from such a visual comparison are similar – quality of Axioplan virtual slides is generally better than quality of corresponding Mirax virtual slides.

In further work, this method could be tested on bigger sets of virtual slides, acquired from different scanners. Also other measures, probably more accurate and efficient, could be proposed. Finally, Haralick features calculated from GLCM could be used for automatic detection of common areas in the virtual slides, so that interaction with user would be minimized.

## Conclusions

In this paper we proposed a method for automatic evaluation of quality of virtual slides which were created by capturing the same glass slide using different scanning devices. The method allows to select common areas of the virtual slides and then chooses fragments of these areas which are analyzed. Quality is evaluated and compared by acquiring Gray-Level Co-occurrence Matrices from the fragments and aggregating Haralick features calculated from the matrices, like contrast or entropy. The method was implemented and tested on two sets of virtual slides, captured by two different scanning devices. The results of the experiment are consistent with opinions of the pathologists about virtual slides’ quality and with visual assessment of quality of the selected fragments.

## Competing interests

The authors declare that they have no competing interests.

## Authors' contributions

SW designed, implemented and tested the method of quality evaluation. JS acquired and preprocessed the sets of virtual slides, on which the method was tested, and compared the tests’ results with visual assessment of quality of virtual slides. All authors read and approved the final manuscript.
